# Hepcidin and Host Defense against Infectious Diseases

**DOI:** 10.1371/journal.ppat.1004998

**Published:** 2015-08-20

**Authors:** Kathryn Michels, Elizabeta Nemeth, Tomas Ganz, Borna Mehrad

**Affiliations:** 1 Departments of Microbiology, Immunology, and Cancer Biology, University of Virginia, Charlottesville, Virginia, United States of America; 2 Department of Medicine, David Geffen School of Medicine at University of California, Los Angeles, Los Angeles, California, United States of America; 3 Department of Pathology, David Geffen School of Medicine at University of California, Los Angeles, Los Angeles, California, United States of America; 4 The Carter Center for Immunology, University of Virginia, Charlottesville, Virginia, United States of America; 5 Department of Internal Medicine, University of Virginia, Charlottesville, Virginia, United States of America; Stony Brook University, UNITED STATES

## Abstract

Hepcidin is the master regulator of iron homeostasis in vertebrates. The synthesis of hepcidin is induced by systemic iron levels and by inflammatory stimuli. While the role of hepcidin in iron regulation is well established, its contribution to host defense is emerging as complex and multifaceted. In this review, we summarize the literature on the role of hepcidin as a mediator of antimicrobial immunity. Hepcidin induction during infection causes depletion of extracellular iron, which is thought to be a general defense mechanism against many infections by withholding iron from invading pathogens. Conversely, by promoting iron sequestration in macrophages, hepcidin may be detrimental to cellular defense against certain intracellular infections, although critical in vivo studies are needed to confirm this concept. It is not yet clear whether hepcidin exerts any iron-independent effects on host defenses.

## Introduction

Iron is necessary for the function of many proteins, including hemoglobin, myoglobin, and enzymes involved in oxidative phosphorylation, and is therefore essential to the survival of virtually all organisms. On the other hand, ionic iron is toxic because of its reactivity with oxygen in the so-called Fenton reaction, which generates the hydroxyl radical and other reactive oxygen species. As an element that is both essential and dangerous, iron availability is tightly regulated. The vast majority of iron is associated with proteins in biological systems, with free iron ions present in extremely low concentrations [1]. In multicellular organisms, this low availability of iron imposes a severe nutritional restriction on invading microbes, and hosts have evolved methods to further limit iron availability in the context of infection. Conversely, professional pathogens have evolved strategies to scavenge iron from the iron-binding proteins of the hosts [2]. Multiple lines of evidence suggest that this battle for iron is a critical component of antimicrobial defenses in many infections [3].

The peptide hepcidin is the master regulator of iron homeostasis in vertebrates [4–6]. Hepcidin was first described as a cationic antimicrobial peptide with microbicidal properties against many microorganisms in vitro [5,7]. Hepcidin is strongly induced during inflammation [8], and emerging data support its role in the pathogenesis of a number of infections. In this article, we review the literature on the role of hepcidin in the resistance and susceptibility to infectious diseases.

### Hepcidin, inflammation, and the regulation of systemic iron availability

**Fig 1:**
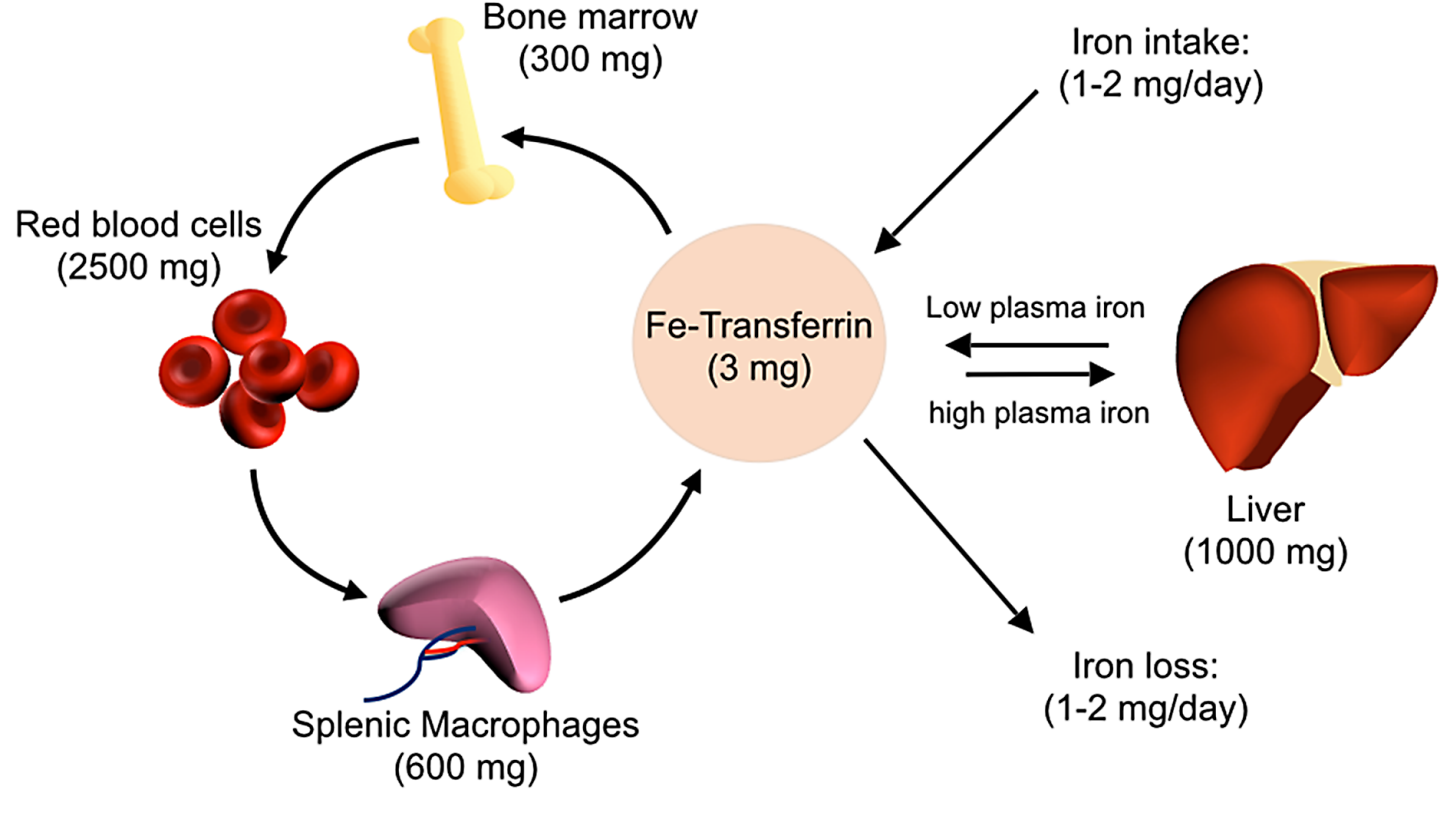
Overview of host iron homeostasis. Iron is absorbed from the diet by duodenal enterocytes and transported into the bloodstream, where it is bound by transferrin. Most iron is incorporated into erythrocytes for heme synthesis. Splenic macrophages recover iron from senescent erythrocytes and release iron into circulation via ferroportin. Smaller amounts of iron are imported into other tissues as needed. Iron loss is not directly regulated and occurs through minor bleeding and shedding of duodenal enterocytes. Approximate iron content of adult human tissues is represented in parentheses.

The global distribution of iron in mammalian hosts is depicted in Fig 1. The majority of the iron is in hemoglobin of circulating erythrocytes and bone marrow erythroid precursors and in splenic and hepatic macrophages that process senescent red blood cells. Hepatocytes serve as an important reservoir that stores or releases iron to maintain homeostasis. Extracellular iron, which constitutes a small proportion of total body iron, is transported in association with the protein transferrin. Stores of intracellular iron are maintained in association with ferritin. During steady state, little iron is absorbed from the diet or lost, with most of the iron requirement being met by recycling iron from red blood cell turnover.

**Fig 2:**
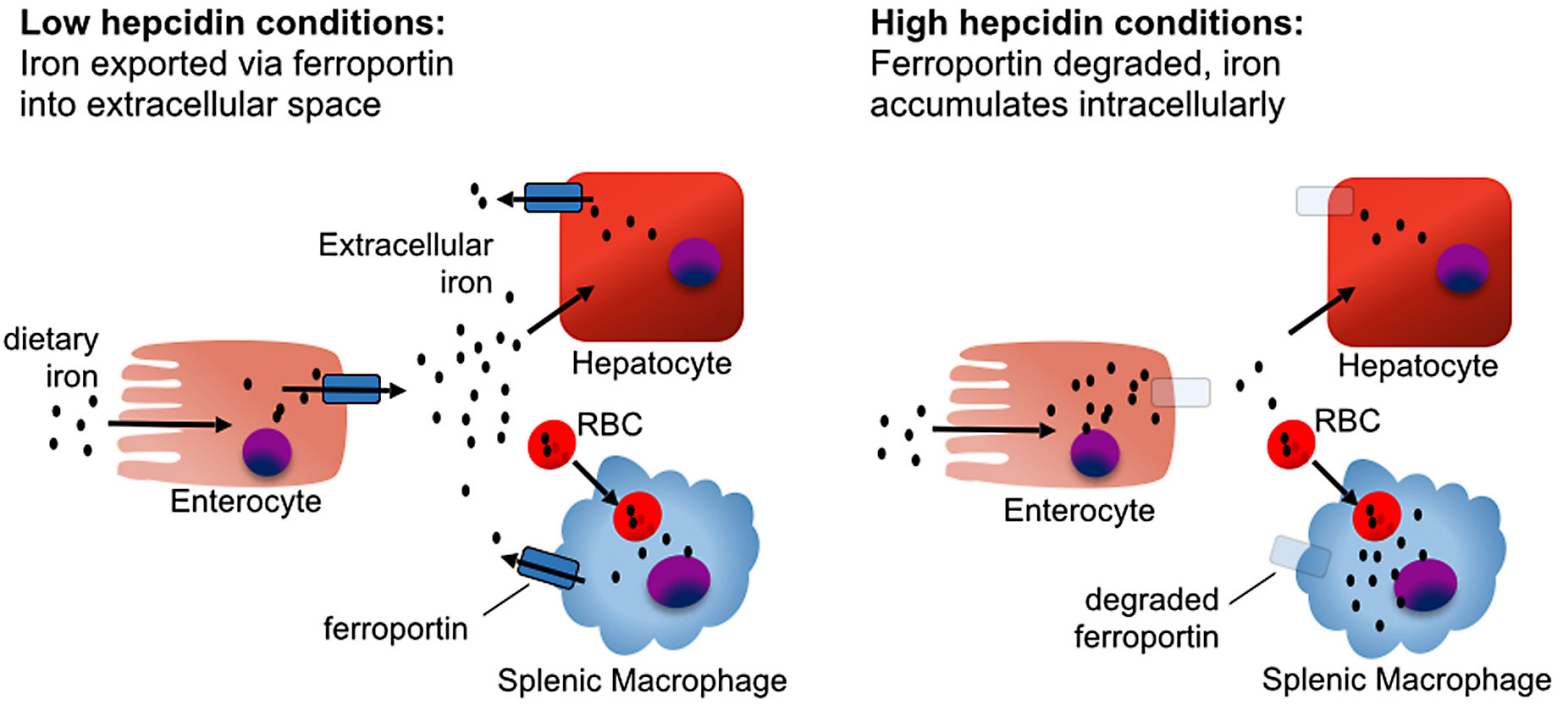
The effect of hepcidin on iron homeostasis. In the absence of hepcidin, iron absorbed from the diet by duodenal enterocytes is transported into the serum via ferroportin, and iron captured from senescent red blood cells is exported from splenic macrophages. In the presence of hepcidin, iron is retained in duodenal enterocytes, which eventually shed from the intestinal tract, blocking iron absorption from the diet. Mononuclear phagocytes retain and accumulate recycled iron rather than releasing it back into circulation, causing a drop in serum iron levels.

The hepcidin–ferroportin axis controls both extracellular iron concentrations and total body iron levels. Ferroportin is a membrane protein that is the major exporter of iron from mammalian cells, including macrophages that recycle iron, duodenal enterocytes that absorb iron, and hepatocytes that store iron. Hepcidin limits the pool of extracellular iron by binding ferroportin and mediating its degradation, thus preventing iron release from intracellular sources (Fig 2) [9]. Sustained elevations of hepcidin result in insufficient iron availability for erythropoiesis, causing an iron-restricted anemia [10]. In contrast, inability to produce or respond to hepcidin causes hereditary hemochromatosis, a group of iron overload disorders resulting from excessive dietary iron absorption and inability to sequester iron in macrophages [4].

**Fig 3:**
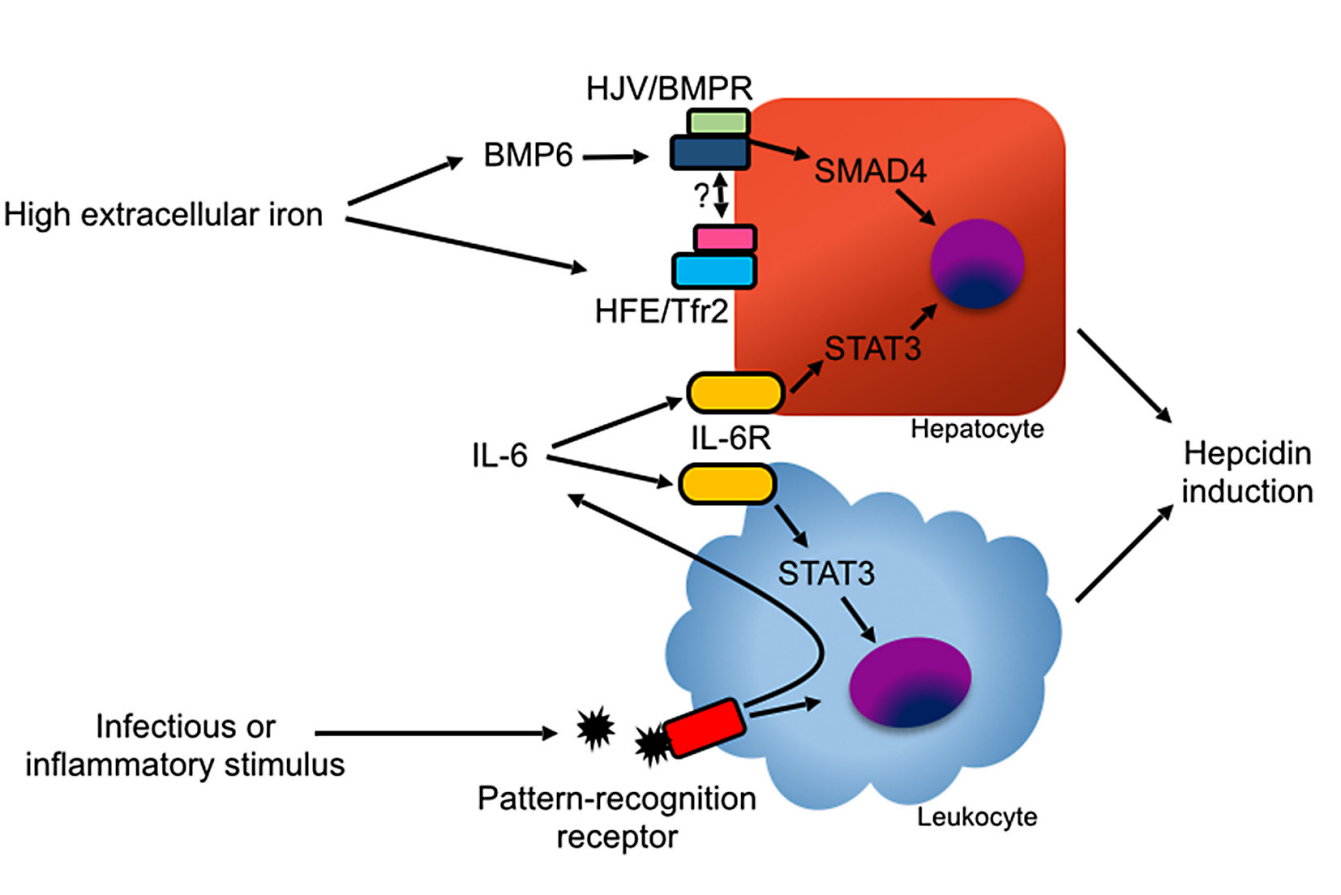
Mechanisms of hepcidin induction. In hepatocytes, hepcidin induction is mediated primarily by BMP ligands binding with the HJV/BMPR complex. BMP6 is induced by high iron levels via an undefined mechanism. The protease TMPRSS6 inhibits hepcidin production by degrading HJV in response to low iron levels [95]. High holo-transferrin levels stabilize the transferrin receptor 2 (Tfr2)/HFE complex, which promotes hepcidin induction, possibly by direct binding with HJV or BMPR [100]. Hepcidin can also be induced by IL-6 via STAT3 signaling in hepatocytes and myeloid leukocytes. Inflammation can stimulate hepcidin production in myeloid leukocytes through pathogen recognition receptor signaling and through autocrine and paracrine production of IL-6.

Most hepcidin is synthesized by hepatocytes. Hepatocyte hepcidin expression is stimulated by elevated extracellular or stored iron and, independently, also by inflammatory stimuli. Conversely, hepcidin expression is inhibited by hypoxia and erythropoiesis [11,12]. Regulation of hepcidin by iron depends on the hemojuvelin (HJV) and bone morphogenetic protein receptor (BMPR) complex activating the SMAD signaling pathway (Fig 3) [13–15]. The study of hepcidin regulation in mice housed under standard conditions is confounded by the high iron content of most commercial mouse chow blends. For mice, an iron-sufficient diet contains approximately 35 parts per million (ppm) iron, while commercial feed contains 150–300 ppm iron, resulting in high basal hepcidin expression [16].

During inflammatory states, hepcidin expression is induced by the cytokine IL-6 via the Janus kinase (JAK) signal transducer and activator of transcription (STAT) 3 pathway [17]. Bone morphogenetic protein (BMP) signaling is also necessary for hepcidin induction during inflammation [18]. In animal models, diverse inflammatory and infectious stimuli, including *Streptococcus* species, *Pseudomonas aeruginosa*, *Aspergillus fumigatus*, influenza, turpentine, and lipopolysaccharide (LPS) robustly induce hepcidin in the liver via the induction of IL-6, resulting in rapid reduction in serum iron levels [16,19–21]. Sustained elevation of hepcidin expression, as occurs in many inflammatory states, results in anemia due to reduced availability of iron for erythropoiesis, a condition previously known as “anemia of chronic disease” and more accurately renamed as “anemia of inflammation” [16,22,23].

In addition to hepatocytes, many cell types, including myeloid leukocytes, express low levels of hepcidin [24,25]. Phagocyte hepcidin expression can be induced by autocrine and paracrine production of IL-6 and, at least in vitro, also by direct engagement of pathogen recognition receptors [24,26–29]. Hepatocyte-specific deletion of hepcidin recapitulates the hemochromatosis phenotype of global hepcidin-deficient animals, indicating that hepatocyte-derived hepcidin is necessary for iron homeostasis in steady state [30]. The role of leukocyte-derived hepcidin has not been formally examined but is hypothesized to contribute to host defense [31,32].

## The Role of Hepcidin in Specific Infections

### Intracellular infections

Hepcidin causes accumulation of iron within cells of the mononuclear phagocyte system, potentially benefiting pathogens that occupy this niche. Type I hemochromatosis, the most common form of hereditary hemochromatosis, is caused by loss-of-function mutations in the *Hfe* gene. HFE protein regulates hepcidin expression in response to increased extracellular iron. Loss of *Hfe* decreases hepcidin expression, increases iron absorption and extracellular iron concentrations, and releases iron from macrophages. *Hfe* mutations have arisen independently in several populations [33,34], and the most common mutation, C282Y, has a heterozygous prevalence of up to 10% in northern European populations [35]. Although difficult to test, it is hypothesized that *Hfe* mutations became prevalent by conferring a survival benefit during population bottlenecks caused by host-adapted pathogens that reside within macrophages as part of their life cycle [36–38].

#### Salmonellosis

Although in vitro data generally support the role of hepcidin in promoting *Salmonella* growth in macrophages, in vivo studies have provided conflicting results. In macrophages experimentally infected with *Salmonella enterica* serovar Typhimurium in vitro, hepcidin expression is induced via autocrine or paracrine mechanisms, causing intracellular iron accumulation and allowing for greater bacterial growth. In vitro infection of murine macrophages with *Salmonella* also resulted in lower intracellular bacterial burdens when macrophages were transfected to overexpress ferroportin [39,40]. In addition, hepcidin treatment increased bacterial burden in cells expressing wild-type, but not hepcidin-resistant, ferroportin [39]. Similarly, elicited peritoneal macrophages from *Hfe*-deficient mice had lower iron content and, when infected with *S*. Typhimurium in vitro, yielded fewer bacteria than wild-type cells [40].

In vivo, mice develop increased hepcidin levels and hypoferremia after oral infection with *S*. Typhimurium [41]. This induction was proposed to be detrimental to the host because prevention of hepcidin induction by an inverse agonist of estrogen-related receptor gamma was associated with improved mouse survival after *S*. Typhimurium infection. In a different study, however, opposite results were reported. Intravenous infection with *S*. Typhimurium did not increase hepcidin mRNA levels in the liver, and hepcidin-deficient mice were more susceptible to infection than their wild-type counterparts [42]. The literature on the susceptibility of *Hfe*-deficient mice to salmonellosis is also contradictory. *Hfe*-deficient mice have been reported to have attenuated intestinal inflammation but higher fecal and systemic bacterial burdens after oral infection in studies that used streptomycin-pretreated mice [43]. In other studies, *Hfe*-deficient mice were reported to have reduced death and bacterial burdens after intraperitoneal inoculation of *S*. Typhimurium that was ascribed to the greater production of lipocalin-2 in *Hfe-/-* macrophages [40].

The reasons for the discrepancies between in vivo studies of salmonellosis are unclear, but may relate to factors such as the age and diet of the experimental animals, which could influence the extent of iron overload at the time of infection and potentially alter the pathogenicity of bacteria. Furthermore, extrapolation of data from *Hfe*-deficient animals to the role of hepcidin is not straightforward, since hepcidin expression in *Hfe*-deficient mice is not abrogated, but only attenuated, relative to degree of iron overload [44]. In addition, there are conflicting reports as to whether *Hfe* influences hepcidin induction to inflammatory stimuli [21,44–46]. Finally, outcomes may be affected by variations in the extracellular portion of the bacterial life cycle, depending on the route of infection.

#### Mycobacterial infections

African iron overload, a condition caused by a combination of high dietary iron intake and non-*Hfe* hereditary hemochromatosis, is strongly associated with death from tuberculosis [47,48], potentially implicating iron homeostasis in host defense against mycobacteria. Consistent with this, macrophages from humans with type I hemochromatosis have lower iron accumulation and lower bacterial burden when infected with *Mycobacterium tuberculosis* as compared to cells from healthy donors; conversely, mycobacteria acquire iron more efficiently from macrophages of healthy subjects as compared to cells from patients with hemochromatosis [49,50]. These data suggest that hepcidin-mediated increase in intracellular iron may be harmful to the host during mycobacterial infections, but this hypothesis has not been addressed directly in vivo. On the other hand, *Hfe*-deficient mice with iron overload had increased tissue bacterial burden after intravenous infection with *M*. *avium* compared to wild-type animals [51], an unexpected result for animals predicted to have iron-depleted macrophages. As with studies in salmonellosis, however, it is difficult to extrapolate data from the *Hfe* mouse model to the role of hepcidin during the infection.

The direct roles of hepcidin and ferroportin in mycobacteria have only been assessed in vitro. Ferroportin overexpressing macrophages have lower mycobacterial burden as compared to normal macrophages when infected with *M*. *tuberculosis* in vitro [52]. Interestingly, ferroportin overexpressing macrophages also have reduced inducible nitric oxide synthase (iNOS) production and phagocytic ability, suggesting that depletion of intracellular iron stores may interfere with macrophage effector functions [52]. Exposure of macrophages to mycobacteria and IFN-γ synergistically induce the expression of hepcidin in vitro, and macrophage-derived hepcidin colocalizes with *M*. *tuberculosis* in coincubation studies [32,53], but the relevance of macrophage-derived hepcidin to in vivo host defense has not been evaluated. Similarly, very high concentrations of hepcidin have been shown to inhibit *M*. *tuberculosis* growth in vitro [32], but it is unknown whether these concentrations are relevant to in vivo infections.

#### Other intracellular pathogens

An in vitro study of murine bone marrow-derived macrophages infected with *Leishmania amazonensis* showed that they up-regulate hepcidin in a TLR4-dependent manner, reduce cell surface ferroportin, and accumulate intracellular iron. Macrophages from hepcidin-deficient mice had lower intracellular burden of parasites as compared to wild-type macrophages after in vitro infection, and hepcidin treatment of wild-type macrophages results in higher parasitic burdens [54]. Similar data have been published with macrophages infected with *Chlamydia* and *Legionella* species [55].

### Infections caused by siderophilic bacteria

Individuals with hereditary hemochromatosis are notably susceptible to sepsis caused by specific microorganisms whose pathogenicity is augmented in the presence of free iron. These so-called siderophilic bacteria include some *Vibrio* and *Yersinia* species, and possibly other Gram-negative bacteria, including *Salmonella* and *Escherichia* species [56,57]. Susceptibility to siderophilic infections in hemochromatosis is thought to be mediated by increased availability of extracellular iron, but in observational studies it is difficult to exclude the contribution of other mechanisms, such as possible immune dysfunction as a result of iron overload and chronic liver disease [58].

#### *Vibrio* infection

Wild-type mice rapidly up-regulate hepcidin and develop hypoferremia after subcutaneous infection with *Vibrio vulnificus*, whereas hepcidin-deficient mice exhibited only mild hypoferremia and are much more susceptible to the infection [59]. The susceptibility of hepcidin-deficient mice was associated with high blood and tissue burden of *V*. *vulnificus* and was specifically attributable to high levels of extracellular iron, since hepcidin-deficient mice with low iron stores (but high serum iron) were also susceptible to infection. Treatment of iron-overloaded hepcidin-deficient mice with a hepcidin agonist was sufficient to restore hypoferremia (without changing iron stores) and to protect hepcidin-deficient mice from infection. Ex vivo studies of bacterial growth in sera from treated animals showed that hepcidin agonists prevented the growth of *V*. *vulnificus* by restricting iron availability rather than by direct antimicrobial activity. Hepcidin agonists did not provide any further protection from *V*. *vulnificus* infection in wild-type mice, again arguing against a direct microbicidal effect of these peptides. Overall, these studies point to hepcidin acting by lowering the concentration of iron species that are not bound to transferrin, and therefore available to *V*. *vulnificus* as nutrients and signals for rapid growth.

### Sepsis

Patients presenting with sepsis syndromes have elevated hepcidin levels that fall during recovery, consistent with activation of the acute phase response during sepsis [60]. Whether hepcidin plays any role in modulating the course of sepsis is unknown, although several mouse studies suggested a protective effect. Hepcidin-deficient mice are more susceptible to death from lethal challenge with LPS as compared to wild-type controls, and administration of an unvalidated synthetic hepcidin protected both wild-type and hepcidin-deficient mice against LPS [61,62]. In murine cecal ligation and puncture model of polymicrobial intra-abdominal sepsis, mice treated intravenously with an adenovirus carrying anti-hepcidin shRNA had higher mortality and bacterial burden associated with increased serum iron levels, whereas conditioning mice on a low-iron diet or treating with an iron chelator resulted in protection following anti-hepcidin shRNA treatment [63]. Similarly, mice that received inhaled anti-hepcidin shRNA adenovirus had higher mortality and more severe lung injury after cecal ligation and puncture compared to mice that received control adenovirus [64]. These studies suggest that expression of hepcidin may be protective during sepsis caused by resident gut flora.

### Malaria

In human studies, iron supplementation is associated with increased incidence and severity of malaria in some [65–67], but not all [68,69], studies, whereas dietary iron deficiency is associated with reduced malaria parasitemia and death [70–72]. Iron deficiency also promotes protection against infection with *Plasmodium berghei* in mice [73,74], suggesting a role for hepcidin in this infection. As expected, humans and mice infected with malaria have elevated plasma hepcidin levels, which correlate positively with parasitemia and plasma IL-6 [75–78], but patients with the most severe anemia in the context of *P*. *falciparum* infection have reduced hepcidin levels, perhaps suggesting negative feedback, such as the effect of compensatory erythropoiesis, on hepcidin expression [75]. In vitro, *P*. *falciparum*-infected erythrocytes induce hepcidin mRNA expression in human peripheral blood monocytes and monocyte-derived macrophages in an IL-6 independent but IL-10 dependent manner [79,80], but the relevance of leukocyte-derived hepcidin for host defense is unknown.

Hepcidin has complex effects in malaria [81]. On one hand, by causing iron restriction, elevated hepcidin likely contributes to anemia. On the other hand, hepcidin may have protective effects in mice during experimental malaria [77,78]. Immunoneutralization of hepcidin results in increased parasitemia and death in *P*. *berghei* infection, whereas pretreatment of animals with a hepcidin-expressing lentivirus protected against parasitemia and death as compared to mice treated with a control lentivirus [78]. Increased hepcidin protected hosts with parasitemia against a second malaria infection [77]; such super-infections are associated with increased mortality in endemic areas. Hepcidin was thought to act by causing the movement of iron from hepatocytes that could host superinfecting parasites to macrophages that cannot. In support of this mechanism, transgenic over-expression or administration of hepcidin to infected mice provided protection by reducing the burden of the parasites in the liver. These data suggest that hepcidin protects against malaria by reducing iron availability to parasites.

### Viral infections

While the induction of serum hepcidin has been documented in several human and murine viral infections [19,26,82], relatively little is known about the contribution of hepcidin to pathogenesis of most viral infections. In an interesting study of serial samples, plasma hepcidin induction during early HIV infection correlated with subsequent viral load set point [83], although the mechanism underlying this correlation is unclear.

#### Hepatitis C

Hepatic iron accumulation is common in hepatitis C virus (HCV) infection and contributes both to liver fibrosis and to increased risk of hepatocellular carcinoma [84]. Patients with hepatitis C who have *Hfe* mutations are further predisposed to hepatic iron overload and worse liver fibrosis [85,86]. Although circulating hepcidin levels correlate positively with the severity of iron overload in chronic HCV infection, patients with HCV infection have a relative deficiency of hepcidin: compared to uninfected controls, HCV patients had lower hepcidin levels for given serum ferritin level, suggesting that hepcidin expression may be blunted in infected patients [87–89]. Similarly, hepcidin was not induced even during acute phases of HCV infection [83], unlike most other infections. Consistent with this, in vitro infection of a hepatocellular carcinoma cell line with HCV resulted in suppression of hepcidin transcription that was associated with higher production of reactive oxygen species [90,91]. This effect was attributable to reduced binding of the transcription factor CCAAT/enhancer-binding protein alpha (C/EBPa) to the hepcidin promoter, and both binding and hepcidin expression could be restored by treatment with antioxidants [91]. In the context of animal models, transgenic mice expressing HCV core protein develop increased serum and hepatic iron, but reduced splenic iron over time as compared to wild-type control animals [92]. This phenomenon was associated with suppressed hepcidin expression and higher ferroportin protein in liver, spleen, and duodenum. Primary hepatocytes from transgenic mice that were transfected with a hepcidin promoter and luciferase reporter construct showed lower luciferase activity compared to hepatocytes cultured from control mice, again associated with reduced binding of C/EBPa to the hepcidin promoter and increased levels of reactive oxygen species [92]. Taken together, these experiments suggest that HCV-mediated hepcidin suppression contributes to iron overload and disease pathology in HCV infection. Whether decreased hepcidin levels and consequent tissue iron loading play any role in viral replication is still unknown.

## Iron-Independent Functions of Hepcidin

Some studies have proposed that hepcidin can influence immune responses independent of its role in iron homeostasis, but it remains to be demonstrated whether iron-independent functions of hepcidin have pathophysiological relevance. One study proposed that hepcidin regulates the production of cytokines and dampens inflammatory responses by activation of the Jak2 pathway [61]. However, it was subsequently shown that Jak2 is not activated by hepcidin and does not interact with ferroportin [93], challenging the proposed mechanism.

Another study showed that iron-deficient macrophages had a more pronounced inflammatory response to LPS treatment, and the inflammatory responses could be dampened with hepcidin treatment alone [94]. To confirm the role of hepcidin, mice deficient in the serine protease TMPRSS6 were used, as these animals have iron deficiency due to high constitutive expression of hepcidin [95]. *Tmprss6*-deficient mice demonstrated a blunted inflammatory response to LPS despite their iron-deficient status [94,96], suggesting that hepcidin may modulate inflammatory responses independently of iron. Other studies, however, reached opposite conclusions: iron-depleted macrophages from *Hfe*-deficient mice, which also have low hepcidin, had attenuated inflammatory response to LPS and *Salmonella* [43]. Thus, studies that dissect the separate effects of hepcidin and macrophage iron on inflammation are needed.

Although defensins and hepcidins are structurally distinct, the hepcidin molecule, like defensins, is an amphipathic peptide with a net cationic charge, is rich in cysteine bonds, and has a β-sheet structure [97]. Consistent with this, hepcidin has microbicidal activity against many classes of microbes in vitro, leading to the hypothesis that such a direct antimicrobial effect may be relevant in vivo during infection [5,7,98]. Serum hepcidin concentrations, however, are 1–2 orders of magnitude lower than those required for antimicrobial effects, making it unlikely that hepcidin directly kills pathogens in the bloodstream [59,98]. While local hepcidin production in infected tissue, and by leukocytes in particular, has been reported, it is unknown if local hepcidin concentrations reach antimicrobial concentrations [24,32]. There is currently little evidence in the literature that hepcidin plays a directly antimicrobial role in vivo in mammalian infections.

## Conclusions and Areas for Further Study

Hepcidin is potently increased by inflammation, but the role of hepcidin in innate immunity is only beginning to be understood, as summarized in Table 1. Hepcidin restricts access to extracellular iron, and this form of “nutritional immunity” is important in at least some extracellular bacterial infections. In contrast, hepcidin induces iron accumulation in macrophages and may be detrimental in defense against pathogens that occupy this intracellular niche. This effect has been demonstrated convincingly in vitro but is not supported by in vivo data [32,39–43,49–55]. Interrogating the role of hepcidin in animal models of intracellular infections should further clarify the complex relationship between iron distribution and pathogenesis of such infections in humans.

**Table 1:** Summary of the role of hepcidin in specific infections. Hepcidin-mediated iron restriction is protective against some extracellular infections and potentially detrimental in host defense against pathogens that reside in the intracellular compartment. Hepcidin has complex effects in infection by *Plasmodium* species and HCV.

Location of infection	Pathogen	Purported role of hepcidin	Quality of evidence
Intramacrophage	*S*. Typhimurium *M*. *tuberculosis*, *M*. *avium*	Detrimental, by promoting sequestration of iron in macrophages	In vitro data support proposed mechanism; in vivo data are conflicting [32, 39–43, 49–53]
	*Chlamydia* sp., *Legionella* sp., *L*. *amazonensis*		In vitro data support proposed mechanism; unaddressed in vivo. [54, 55]
Extracellular	Siderophilic bacteremia, polymicrobial sepsis	Protective, via plasma iron restriction; possibly attenuates inflammation	Correlative human data and experimental mouse infection [56, 57, 59, 61–64]
Erythrocytes and hepatocytes	*P*. *falciparum*, *P*. *berghei*	Anemia promotes clearance of infected erythrocytes; hepcidin promotes depletion of iron in hepatocytes	Human correlative data and experimental mouse data [65–80]
Hepatocytes	HCV	HCV infection suppresses hepcidin expression, contributing to iron overload	Correlative human data, experimental mouse data, and in vitro data [82, 83, 85–92]

In the absence of in vivo data, any iron-independent role of hepcidin in host defense remains speculative. In particular, there is little evidence to support a direct microbicidal role for hepcidin in mammalian infections. The current literature suggests that hepcidin may dampen inflammatory cytokines through a mechanism that is not well understood. As excessive inflammation is damaging in many infections, the potential role of hepcidin as a mediator of the innate immune response is a new and unexpected area of study.

The role of hepcidin remains undefined in most infections and awaits further investigation. For example, although hepcidin is induced in response to several viral and fungal pathogens [20,99], its contribution to host defenses against these infections is largely unknown. With the exception of malaria and Leishmaniasis, hepcidin has not been investigated in parasitic infections.
